# Successful Distribution of Tecovirimat During the Peak of the Mpox Outbreak — Los Angeles County, June 2022–January 2023

**DOI:** 10.15585/mmwr,mm7324a2

**Published:** 2024-06-20

**Authors:** Margaret J. O’Neil, Roxanne Archer, Phoebe Danza, Rebecca Fisher, Dee Ann Bagwell, Ibrahim Younis, Sonali Kulkarni, Zachary Rubin, Moon Kim, Sharon Balter, Dawn Terashita, Jee Kim, Rita Singhal, Daniel Hancz, Marianne Gausche-Hill, Naman K. Shah

**Affiliations:** ^1^Division of HIV and STD Programs, Los Angeles County Department of Public Health, Los Angeles, California; ^2^Acute Communicable Disease Control Program, Los Angeles County Department of Public Health, Los Angeles, California; ^3^Bureau of Disease Control, Los Angeles County Department of Public Health, Los Angeles, California; ^4^Emergency Preparedness and Response Division, Los Angeles County Department of Public Health, Los Angeles, California; ^5^Clinic Services, Los Angeles County Department of Public Health, Los Angeles, California; ^6^Emergency Medical Services, Los Angeles County Department of Public Health, Los Angeles, California.

**Summary**

**What is already known about this topic?**

Tecovirimat is recommended for severe mpox under an expanded access investigational new drug protocol. During the 2022–2023 mpox outbreak, local U.S. health jurisdictions facilitated access to tecovirimat.

**What is added by this report?**

Using emergency preparedness plans, Los Angeles County developed a hub and spoke tecovirimat distribution network, facilitating treatment of approximately one third of patients with mpox; most were treated in clinics and pharmacies. The median interval from specimen collection to treatment was 2 days.

**What are the implications for public health practice?**

Medical countermeasures can be deployed during public health emergencies using existing distribution networks and local surveillance data to facilitate treatment access.

## Abstract

Tecovirimat is the first-line antiviral treatment recommended for severe mpox or for persons with mpox who are at risk for severe disease; tecovirimat is available in the United States under an expanded access investigational new drug (IND) protocol. During the 2022–2023 mpox outbreak, local U.S. health jurisdictions facilitated access to tecovirimat. In June 2022, Los Angeles County (LAC) rapidly developed strategies for tecovirimat distribution using existing medical countermeasure distribution networks established by the Public Health Emergency Preparedness Program and the Hospital Preparedness Program, creating a hub and spoke distribution network consisting of 44 hub facilities serving 456 satellite sites across LAC. IND patient intake forms were analyzed to describe mpox patients treated with tecovirimat. Tecovirimat treatment data were matched with case surveillance data to calculate time from specimen collection to patients receiving tecovirimat. Among 2,281 patients with mpox in LAC, 735 (32%) received tecovirimat during June 2022–January 2023. Among treated patients, approximately two thirds (508; 69%) received treatment through community clinics and pharmacies. The median interval from specimen collection to treatment was 2 days (IQR = 0–5 days). Local data collection and analysis helped to minimize gaps in treatment access and facilitated network performance monitoring. During public health emergencies, medical countermeasures can be rapidly deployed across a large jurisdiction using existing distribution networks, including clinics and pharmacies.

## Introduction

In May 2022, clade II mpox, historically endemic in West and Central Africa, became a widespread outbreak, with a public health emergency declared in the United States in August 2022.* Tecovirimat, an antiviral treatment developed to treat smallpox, is a first-line drug for use in patients with severe mpox or those at risk for severe disease (*1*,*2*).

CDC holds an expanded access investigational new drug (IND) protocol for using tecovirimat to treat mpox (*1*). Patients with mpox are eligible for treatment with tecovirimat if they meet IND eligibility criteria, which have evolved since the start of the outbreak^†^ (*1*). At the start of the outbreak, tecovirimat was available through the Strategic National Stockpile (SNS) and was prepositioned for distribution by state and local health departments (*1*).

Los Angeles County (LAC), with 10 million residents in a 4,058–square mile area, was an epicenter of the mpox outbreak at its peak in the United States; LAC experienced the highest California mpox case count, accounting for 39% of all California cases reported by January 30, 2023 (*3*). During June 2022–January 2023, a cross-sectional study of patients with mpox who received tecovirimat by LAC health care providers during the mpox emergency was conducted. This study describes the tecovirimat distribution network in LAC, characteristics of treated patients, and local surveillance data that measured the time to treatment during the mpox public health emergency.

## Methods

The LAC Department of Public Health (DPH) serves all county areas except Pasadena and Long Beach, which have separate health departments. DPH created an mpox treatment unit comprising part-time and full-time clinical and surveillance staff members, with support from existing operations personnel available for after-hours calls, DPH direct clinical care, DPH and independent pharmacy support, and supply management.

### Prepositioning and Distribution of Tecovirimat

Tecovirimat supply from SNS was prepositioned at a DPH warehouse facility. LAC recruited health care providers experienced in distributing medical countermeasures to serve as network hubs; these hubs were responsible for distributing tecovirimat to their affiliates (Supplementary Figure, https://stacks.cdc.gov/view/cdc/157468). Hubs were selected based on geographic location, proximity to patients, number of providers, overall patient volume, and availability of extended hours of operation. Hub providers included community clinics, pharmacies, and LAC’s Disaster Resource Center hospitals and were supplied with tecovirimat and trained on its use, including IND and reporting requirements. Data reported to DPH were reviewed weekly and used to provide supply and to target additional training and site visits. Hubs received technical assistance for incorporating the dispensing of tecovirimat into their specific patient workflows. Disaster Resource Center hospitals received a single course of intravenous tecovirimat and twenty courses of oral tecovirimat to distribute on-demand to their associate general acute care hospitals through the Hospital Preparedness Program. Health care workers at hub affiliates (spokes) were trained to request tecovirimat from their respective hubs, complete the IND processes, and administer the drug. DPH fulfilled hub tecovirimat orders based on clinical volume and existing inventory. For patients with mpox who did not have a medical home or a provider willing to treat them, DPH provided patient navigation services, coordination through provider and patient consult lines, and public health nurse follow-up of all mpox cases. Consultation was available to all providers at any time.

### Data Management

The IND protocol requires intake forms that include demographic information and medical histories for each tecovirimat-treated patient to be sent to CDC. Providers sent a copy of the intake form to DPH, which was entered into a REDCap database (version 14.1.2; Vanderbilt University). Patient intake forms were matched to available mpox case data in DPH’s disease surveillance database using SAS statistical software (version 9.4; SAS Institute). A matching process was used to link tecovirimat-treated patients to available hospitalization records, specimen collection data, laboratory results, and death records. The DPH Institutional Review Board judged this secondary analysis to be exempt from institutional review board approval processes.

## Results

### Tecovirimat Distribution Network

**FIGURE 1:**
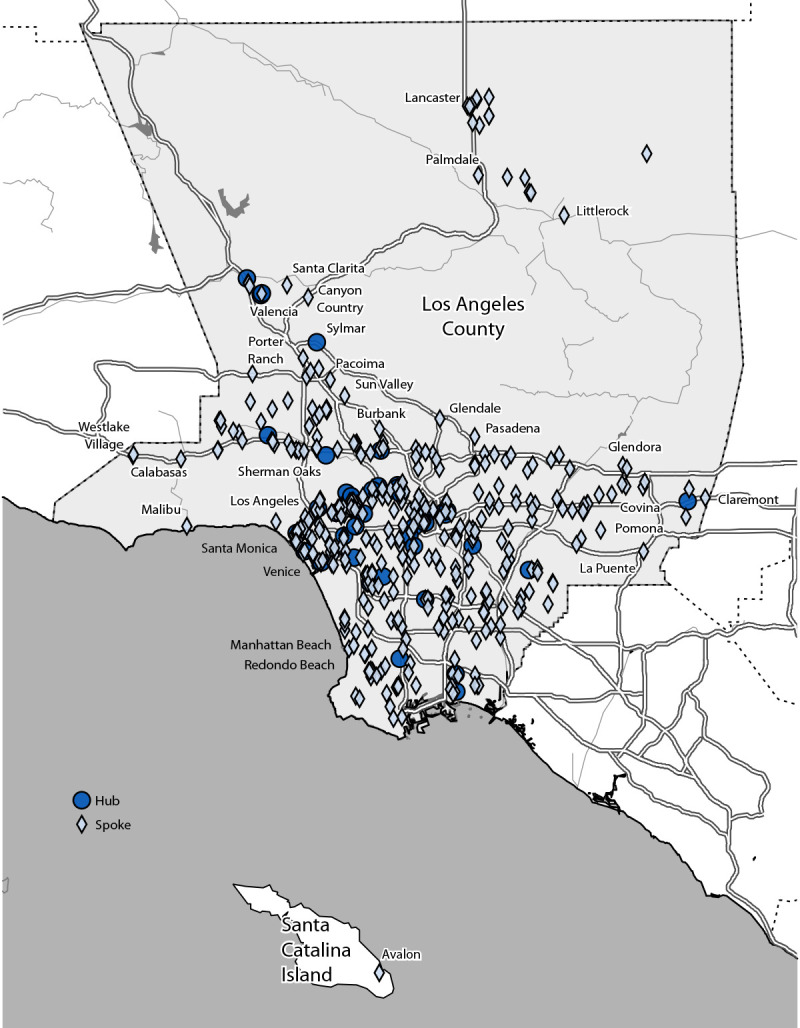
Tecovirimat hub and spoke provider sites — Los Angeles County, California, June 2022–January 2023

By August 1, 2022, the tecovirimat distribution network comprised 44 hub facilities across LAC, including 23 clinics, 11 hospitals, and 10 independent pharmacies (Figure 1). These hubs served 456 associated spoke facilities. By January 31, 2023, among 2,281 patients with mpox in LAC, approximately one third (735; 32%) had been treated with tecovirimat (Table). The patients with mpox who were not treated with tecovirimat (1,546; 68%) likely did not have severe mpox, were not at risk for severe mpox, or experienced barriers to receiving tecovirimat, resulting in missed treatment opportunities. 

**TABLE:** Characteristics of total mpox cases and those treated with tecovirimat — Los Angeles County, California, June 2022–January 2023*

Characteristic	No. (column %)	
	All patients (N = 2,281)	Treated with tecovirimat (n = 735)
**Gender**		
Cisgender male	**2,177 (95)**	659 (90)
Cisgender female	**45 (2)**	11 (2)
Transgender male	**0–10 (<1)**	0–10 (<1)
Transgender female	**28 (1)**	13 (2)
Another gender identity	**23 (1)**	42 (3)
Prefer not to say or unknown	**0–10 (<1)**	NA
**Age group, yrs**		
0–17	**11 (<1)**	0–10 (<1)
18–29	**551 (24)**	104 (14)
30–39	**980 (43)**	317 (43)
40–49	**487 (21)**	200 (27)
50–59	**208 (9)**	93 (13)
≥60	**44 (2)**	12 (2)
Unknown	**0 (—)**	0–10 (<1)
**Race and ethnicity^†^**		
American Indian or Alaska Native	**12 (1)**	0–10 (<1)
Asian	**70 (3)**	24 (3)
Black or African American	**351 (15)**	109 (15)
Native Hawaiian or Pacific Islander	**0–10 (<1)**	0–10 (<1)
White	**567 (25)**	190 (26)
Hispanic or Latino	**1,085 (48)**	298 (41)
Multiple races	**26 (1)**	NA
Other	**33 (1)**	35 (5)
Unknown	**133 (6)**	74 (10)
**HIV-positive**	**1,026 (45)**	**375 (51)**

**Abbreviation:** NA = not available.

* Case counts between zero and 10 are suppressed to prevent possible identification based on Los Angeles County Department of Public Health Chief Science Office guidelines.

^†^ Persons of Hispanic or Latino (Hispanic) origin might be of any race but are categorized as Hispanic; all racial groups are non-Hispanic.

**FIGURE 2:**
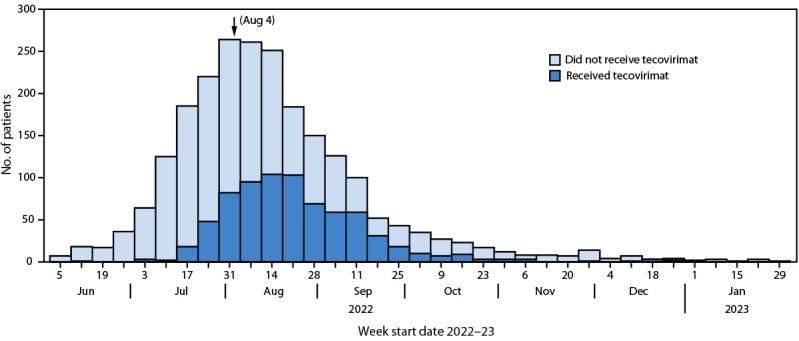
Number of patients with mpox who received and did not receive tecovirimat, by week (N = 2,281)*^,^^†^ — Los Angeles County, California, June 2022–January 2023 * A total of 1,546 patients with mpox did not receive tecovirimat; 735 received tecovirimat. ^†^ Public health emergency was declared in the United States on August 4, 2022, in response to the mpox outbreak.

Overall, 120 (16%) patients who received tecovirimat were treated at pharmacies, 227 (31%) at hospitals, and 388 (53%) at community clinics. The majority of (685; 93%) patients were treated in outpatient settings; only 48 (7%) received inpatient treatment. Seven (1%) patients received intravenous tecovirimat, and the remaining 728 (99%) received oral tecovirimat. The peak treatment period occurred in August 2022 (423; 58%) after the peak in confirmed mpox cases in late July (Figure 2).

### Characteristics of Tecovirimat-Treated Patients with Mpox

Among the 735 patients with mpox in LAC who received tecovirimat during June 2022–January 2023, gender was known for 670 (91%), 659 (90%) of whom identified as male (Table). The median patient age was 38 years (range = 9–79 years). Clinician-reported reasons for tecovirimat treatment were lesions in anatomic areas that might result in serious sequelae (549; 75%), pain (404; 55%), and risk for severe outcomes due to immunosuppression (229; 31%); one patient was pregnant. Overall, 375 (51%) patients reported HIV infection as a medical comorbidity. Among all tecovirimat-treated patients, 333 (45%) had 10–100 lesions.

### Interval from Diagnosis to Treatment, Empirical Treatment, and Patient Outcomes

Among the 525 (71%) treated patients who were matched to case surveillance records, the median interval from specimen collection or presumptive diagnosis to receipt of dispensed tecovirimat was 2 days (IQR = 0–5 days); this finding did not vary by month from June 2022 through January 2023. The median was 4 days (IQR = 0–5 days) for patients receiving dispensed tecovirimat after laboratory confirmation compared with zero days for those who received dispensed tecovirimat empirically. Among the 485 patients with matched records for whom mpox test results were available, 307 (63%) were treated after receiving a confirmed diagnosis, whereas 155 (37%) were treated empirically; three (2%) of these persons ultimately received a negative mpox test result. Overall, 47 (9%) treated patients with matched surveillance records were hospitalized, and two deaths (0.4%) were reported.

## Discussion

DPH streamlined tecovirimat distribution using a hub and spoke distribution model, facilitating provision of tecovirimat to multiple access points. Combining patient intake forms with provider tecovirimat inventory and overall trend data allowed DPH to ascertain supply sufficiency and conduct outreach to restock, preventing access gaps. Whereas resources for emergency preparedness planning, including the distribution of medical countermeasures, have focused on hospitals (*4*), in this setting, approximately two thirds of treated patients received tecovirimat at clinics and pharmacies. This strategy was built on the COVID-19 vaccine rollout, for which clinics and pharmacies were crucial to vaccine distribution networks (*5*).

Throughout the emergency and during changes in protocol, tecovirimat use rates paralleled disease incidence (*6*). The proportion of reported patients with mpox treated with tecovirimat in LAC (33%) was higher than that reported nationally (23%); however, national data might reflect underreporting (*7*). Other reasons might be that reported patients with mpox treated with tecovirimat in LAC represent a larger proportion of reported patients with severe mpox disease or patients at risk for severe disease, or that patients with treatment indications had better access to treatment. Although tecovirimat treatment training balanced access to care with judicious use to minimize the risk for emergence of tecovirimat resistance, the higher proportion of treated patients could also represent unnecessary antiviral treatment resulting from subjective clinician assessment. The median interval from specimen collection to receipt of tecovirimat by patients with mpox in LAC was 2 days. Local public health and commercial laboratory turnaround times were approximately 2 and 3 days, respectively. Overall, the distribution strategy was effective and sufficient to meet increasing needs. Similar strategies might benefit tecovirimat distribution in different jurisdictions as would other prepositioned therapeutics for mpox or future emerging infections.

### Limitations

The findings in this report are subject to at least two limitations. First, assessment of interval from diagnosis to treatment was precluded among patients who received tecovirimat but whose records were not linked with surveillance data; this finding might increase or decrease the reported interval because 29% of tecovirimat-treated patients were not able to be matched to case surveillance data based on the identifying information provided on their patient intake forms. Second, because intake forms were only completed at the beginning of treatment, no reports were required at completion of treatment, and well-defined endpoints for antiviral treatment of mpox were absent, LAC was unable to assess the effectiveness of the distribution system regarding its contribution to a reduction in morbidity and mortality (*8*).

### Implications for Public Health Practice

Local health jurisdictions can rapidly deploy medical countermeasures over a wide area using existing distribution systems created by the Public Health Emergency Preparedness Program and Hospital Preparedness Programs and should include clinics and pharmacies in emergency preparedness planning. Local data collection and analysis can reduce gaps in access to treatment and facilitate monitoring the distribution and program performance. Future preparedness efforts can focus on measuring additional administrative costs associated with a medical countermeasure, such as costs of staff training, work hours, storage of medical countermeasure products, and supporting distribution partners. Accurate data to improve case matching and define and collect outcome data are needed.

## Footnotes

*
https://www.cdc.gov/poxvirus/mpox/response/2022/world-map.html

†
https://www.cdc.gov/poxvirus/mpox/clinicians/obtaining-tecovirimat.html
